# Noninvasive *In Vivo* Quantification of Neutrophil Elastase Activity in Acute Experimental Mouse Lung Injury

**DOI:** 10.1155/2011/581406

**Published:** 2011-09-18

**Authors:** Sylvie Kossodo, Jun Zhang, Kevin Groves, Garry J. Cuneo, Emma Handy, Jeff Morin, Jeannine Delaney, Wael Yared, Milind Rajopadhye, Jeffrey D. Peterson

**Affiliations:** PerkinElmer, 549 Albany Street, Boston, MA 02118, USA

## Abstract

We developed a neutrophil elastase-specific near-infrared fluorescence imaging agent, which, combined with fluorescence molecular tomographic imaging, allowed us to detect and quantify neutrophil elastase activity *in vivo*, in real time, and noninvasively in an acute model of lung injury (ALI). Significantly higher fluorescent signal was quantified in mice with LPS/fMLP-induced ALI as compared to healthy controls, correlating with increases in the number of bronchoalveolar lavage cells, neutrophils, and elastase activity. The agent was significantly activated *ex vivo* in lung sections from ALI but not from control mice, and this activation was ablated by the specific inhibitor sivelestat. Treatment with the specific inhibitor sivelestat significantly reduced lung signal in mice with ALI. These results underscore the unique ability of fluorescence molecular imaging to quantify specific molecular processes in vivo, crucial for understanding the mechanisms underlying disease progression and for assessing and monitoring novel pharmacological interventions.

## 1. Introduction

Acute lung injury (ALI) and its more severe manifestation, acute respiratory distress syndrome (ARDS) are life-threatening conditions caused by a variety of insults such as sepsis, trauma, pneumonia, inhalation of toxic chemicals or fumes, and pulmonary aspiration [1]. In the US there are an estimated yearly 190,000 new cases of ALI and 140,000 of ARDS, with an overall pooled mortality rate of 43%. Each year, cases of ALI alone require 3.6 million hospital days and 2.15 million ICU days, a significant burden in terms of morbidity, mortality, length of hospitalization, and need for rehabilitation [1, 2]. 

Animal studies have shown that both ALI and ARDS are characterized by an alteration of the alveolar capillary barrier which results in fluid-filled airspaces, spread of pathogens, loss of surfactant, and neutrophil infiltration. Activated neutrophils in turn release growth factors, cytokines, reactive oxygen species, and proteases, such as neutrophil elastase (NE), contributing to tissue damage, organ dysfunction, and further exacerbating the inflammatory process [3, 4]. NE, also known as leukocyte elastase or ELA2, is a 30-kD glycoprotein chymotrypsin-like serine protease with broad substrate specificity, capable of degrading many components of the extracellular matrix such as collagen, elastin, fibrin, and fibronectin. NE is stored at high concentrations in neutrophil azurophil granules, together with proteinase 3 (PR3), cathepsin (Cat) G, and matrix metalloproteinase- (MMP-) 9, and is also found at the surface of neutrophils and free in the extracellular milieu [5–7]. Not only is NE a significant protease involved in ALI and ARDS, but also in many other inflammatory processes such as emphysema/chronic obstructive cystic fibrosis, chronic wound healing, rheumatoid arthritis, as well as ischemia-reperfusion, atherosclerosis, septicemia, and pneumonia [3].

Because of the high prevalence of ALI and ARDS, the development of noninvasive techniques to spatiotemporally visualize and quantify disease-associated NE *in vivo* is an active area of research in many laboratories around the world. Advances in optical imaging techniques will potentially provide new tools for understanding the roles of NE in disease onset and progression, as well as in the development and assessment of specific NE inhibiting therapies. Clinical applications of NE imaging may also help in ALI/ARDS diagnosis, staging of disease, and monitoring of treatment efficacy, as well as in assessing acute neutrophilia at other sites of infection and inflammation. For example, a radiolabeled aptamer-based inhibitor of NE coupled to ^99m^Tc has been used to image inflammation in a rat reverse passive Arthus reaction model [8] and a ^99m^Tc-labeled peptide NE inhibitor was used to visualize inflammation and infection in rhesus monkeys [9]. While established noninvasive imaging modalities like PET and SPECT are sensitive and provide functional information, their dependence on ionizing radiation (with its related costs, complexity, shorter half-lives, and radioactive material handling/disposal) limit their use. Fluorescence offers an alternative to the use of radiolabels, but until recently fluorescence detection was limited to the assessment of surface fluorescence by reflectance imaging. Recent advances in optical imaging led to the development of fluorescence molecular tomography (FMT) [10, 11] imaging which, paired with appropriate near infrared imaging agents, has been used for imaging and quantification of numerous biological targets in 3 dimensions [12, 13]. FMT has been used recently for imaging protease activity associated with lung inflammation and treatment efficacy in mouse models of asthma [11, 14, 15], highlighting its capabilities in deep tissue detection. 

The present studies were undertaken to develop and validate a novel NE-selective activatable near-infrared fluorescent (NIRF) agent, Neutrophil Elastase 680 FAST (NE680), for use in imaging and quantifying NE activity in mouse models of neutrophil-mediated inflammation. Specificity of the agent was confirmed by screening with a panel of related, and unrelated, proteases and by inhibition using the specific NE inhibitor *in vitro, in vivo*, and in *ex vivo* tissue sections. Most importantly, NE680 was used *in vivo* to image and quantify NE activity associated with lung inflammation in mice with ALI and response to treatment. 

## 2. Materials and Methods

### 2.1. Fluorogenic Neutrophil Elastase 680 FAST Agent

The fluorogenic NE680 was provided by PerkinElmer (Neutrophil Elastase 680 FAST, Boston MA). Briefly, two NIR fluorochromes (VivoTag-S680, PerkinElmer, Boston, MA) were linked to both the C- and N-termini of the peptide PMAVVQSVP, a highly NE-selective sequence over mouse PR3 [16]. The substrate was further conjugated to a polymer carrier at a ratio of 1 substrate per polymer molecule giving the agent a final molecular weight of approximately 40,000 daltons. UV-Vis absorbance and fluorescence emission spectra of the native and enzyme-activated agent were recorded on a Cary 50 and Cary Eclipse spectrophotometers, respectively, in 1× PBS using 665 nm for fluorescence excitation.

### 2.2. Mouse Plasma Stability

The agent was incubated in normal mouse plasma (Innovative Research, Novi, MI) diluted 1 : 4 in PBS, pH 7.40 with 1 mM EDTA, at 37°C for 24 h. The stability of the agent was analyzed by HPLC.

### 2.3. Pharmacokinetics

Twenty-four female retired breeder CD-1 mice (age 12–16 weeks, Charles River Laboratories, Wilmington, MA) received a bolus intravenous (i.v.) injection of NE680 (2 nmol in PBS). Terminal blood samples (*n* = 3 mice per time point) were collected by cardiac puncture from each mouse (following carbon dioxide asphyxiation). Plasma was collected by centrifugation (15,000 rpm for 10 min at 4°C) in EDTA-containing tubes. Aliquots (50 *μ*L) of each plasma sample were placed in Eppendorf tubes. Cold methanol (150 *μ*L) was added and the tubes were vortexed followed by centrifugation at 12,000 rpm and 4°C for 10 minutes. Approximately 110 *μ*L of the supernatants were transferred to HPLC vials for analysis. Studies were conducted in accordance with and approved by PerkinElmer's IACUC Institutional Animal Care and Use Committee guidelines.

### 2.4. Agent Characterization by HPLC

HPLC analyses were performed on a Waters model 2695 (Waters Corporation, Milford, MA). The PDA, Waters model 2998, was set to scan from 225 nm to 800 nm. The wavelength corresponding to the absorbance maximum of the fluorophore, 675 nm, was extracted from the PDA trace. The fluorescence spectrophotometer was set to an excitation wavelength of 675 nm and emission was monitored at 693 nm. Samples were analyzed on a C4, 300 Å, 5 *μ*m, 150 × 4.6 mm HPLC column (Phenomenex, Torrance, CA). The aqueous mobile phase contained 25 mM ammonium formate, pH 8.5. Samples were eluted with acetonitrile at a flow rate of 1 mL/min. A gradient of 15% to 85% organic provided sufficient resolution. Standards were prepared with NE680 (0–2.5 *μ*M) in mouse plasma. Standard curves had correlation coefficients >0.98.

### 2.5. *In Vitro* Activation by Neutrophil Elastase and Related Enzymes

Activation and protease selectivity of NE680 were determined with NE and closely related enzymes including PR3, Cat G, Cat B, and MMP-9. The assays were performed with 0.05 *μ*M of enzyme and 0.5 *μ*M NE680 in the optimized buffer and pH for each enzyme. Human NE was purchased from Innovative Research (Novi, MI), recombinant mouse NE, mouse Cat B, and active mouse Cat C from R and D Systems (Minneapolis, MN), human neutrophil PR3 from Athens Research and Technology (Athens, GA), and human neutrophil Cat G and recombinant human MMP-9 from BIOMOL International (Plymouth Meeting, PA). The reaction buffers were as follows: for human NE, 100 mM Tris (pH 7.5); for mouse NE, 50 mM Tris (pH 7.5), 1 M NaCl, 0.05% Brij-35; for Cat B, 25 mM MES (pH 5.0), 0.5 mM DTT (preactivation in 25 mM MES pH 5.0, 5 mM DTT for 15 min at room temperature); for MMP-9, 50 mM Tris (pH 7.5), 10 mM CaCl_2_, 150 mM NaCl, 0.05% Brij 35; for Cat G, 100 mM Tris (pH 7.5), 1.6 M NaCl; for human PR3, 100 mM Tris (pH 7.5), 500 mM NaCl. Mouse NE was activated by active mouse Cat C in 50 mM MES, 50 mM NaCl, pH5.5 at 37°C for 2 h. Reactions were carried out at room temperature in 250 *μ*L in 96 well plates with black sides and bottom. All the reactions were monitored at various time points at excitation/emission wavelengths of 663/690 nm with a cutoff at 665 nm using a fluorescence plate reader (Molecular Devices, San Leandro, CA). The released fluorescence is shown after subtracting background fluorescence of the agent without enzyme.

### 2.6. Inhibition of NE by Sivelestat

To confirm the specificity of NE680 activation by NE and not other proteases, in vitro, ex vivo, and in vivo studies were performed in the absence or presence of sivelestat (N-{2-[({4-[(2,2-dimethylpropanoyl) oxy]phenyl}sulfonyl) amino]benzoyl} glycine sodium salt; Tocris Bioscience, Ellisville, MO), a well-described specific NE inhibitor [17]. The IC_50_ against human NE and human PR3 were determined as described above using NE680 (0.5 *μ*M). Reactions were performed at room temperature with a 30 min preincubation of human NE or PR3 with serially diluted sivelestat. The IC_50_ represents the concentration needed to achieve 50% inhibition of agent cleavage.

### 2.7. Acute Lung Injury Mouse Model

Acute lung inflammation was induced in mice according to published protocols with slight modifications [18, 19]. Male CD-1 mice were purchased from Charles River Laboratories (Wilmington, MA) and used when they reached 8–10 weeks of age. Mice were housed in environmentally controlled specific-pathogen-free conditions with water and low-fluorescence mouse chow (Harlan Teklad, Madison, WI). All animal experimental procedures were approved by PerkinElmer's Institutional Animal Care and Use Committee and were in accordance with veterinarian requirements. On day 1, mice were challenged intranasally (i.n.) with 100 *μ*g of LPS (E. coli 111 : B4, Sigma, St. Louis, MO) solubilized in 40 *μ*L PBS or PBS only. Eighteen hours later, mice received an intranasal instillation of the chemotactic peptide N-formyl-met-leu-phe (fMLP, Sigma, St. Louis, MO) at 200 nM in 40 *μ*L PBS together with 4 nmoles of NE680. To analyze the relative contribution of NE to the activation of NE680 *in vivo*, groups of mice were treated i.n. with or without NE inhibitor sivelestat (5 mg/kg 15 min before fMLP and NE680 delivery).

### 2.8. Bronchoalveolar Lavage and Lung Collection

To validate the model, mice which did not receive NE680, were sacrificed after LPS (23 h) and fMLP (5 h) challenge, and the tracheas were exposed through a midline incision. A sterile 22-gauge needle connected to a 1 mL syringe was inserted and used to lavage the lungs *in situ *with a total of 1 mL PBS. The bronchoalveolar lavage (BAL) thus obtained was centrifuged at 1000 rpm for 5 min. The pelleted cells were counted and cell types were analyzed under microscopy after cytospinning at 700 rpm for 7 min (Shandon Scientific) onto glass slides, fixing in methanol and staining with Giemsa for 5 min. The remaining BAL fluids (BALF) were kept at −20°C until used for NE activity assays. After lavage, the lungs were removed, rinsed with sterile saline, and homogenized at a weight : volume ratio of 140 mg per mL of 0.1 M Tris pH 7.4. After 3 cycles of freezing and thawing the lung lysates were centrifuged at 14,000 for 30 min at 4°C. Supernatants were kept at −20°C until use for NE assays.

### 2.9. Neutrophil Elastase In Vitro Assays

NE activity in the BALF and lung homogenates was measured using the well-established MeOSu-AAPV-AMC substrate. One hundred *μ*L of BALF or lung lysates were incubated with MeOSu-AAPV-AMC (0.1 nM final concentration) in 0.1 M Tris pH 7. Reactions were carried out in 250 *μ*L in 96 well plates with black sides and bottom. All the reactions were monitored at various timepoints at excitation/emission wavelengths of 400/505 nm with a cutoff at 450 nm using a fluorescence plate reader (Molecular Devices, San Leandro, CA). The released fluorescence is shown after subtracting background fluorescence of the substrate only.

### 2.10. NE680 Activation in Lung Sections

To validate the specificity of NE680 activation by NE present in lungs, mice with lung inflammation and control healthy mice (which had not received NE680) were euthanized by CO_2_ inhalation and lungs were removed and snap frozen in OCT. Ten *μ*m lung sections were incubated with 1 *μ*M NE680 in the absence or presence of increasing concentrations of sivelestat (0.04–4 *μ*M) at 37°C in a humidified incubator for 5 h. Fluorescence, as a measure of agent activation, was captured under fluorescence microscopy using appropriate filters (Zeiss Axioskop 2 MOT Plus). DAPI was used as a nuclear counterstain. 

### 2.11. *In Vivo* Imaging and Analysis

For *in vivo* imaging, mice were first depilated using Nair and placed in a fluorescence molecular tomography (FMT) 2500 system (PerkinElmer, Boston, MA) imaging chamber to which is delivered a controlled amount of isoflurane/oxygen mixture keeping the mice anesthetized. Three-dimensional regions of interest (ROIs) were drawn around the chest area applying a universal threshold, equal to 40% of the mean concentration value of fluorescence in the LPS/fMLP mice (in nM). The total amount (in picomole) of fluorochrome was automatically calculated relative to internal standards generated with known concentrations of the appropriate fluorochrome. For each study, the mean fluorescence of the LPS/fMLP group was equaled to 100%, and then each mouse in that study was normalized accordingly. Shown are the grouped results of 3 studies, as the percentage of LPS/fMLP Lung Fluorescence (means ± S.E.M.).

### 2.12. Fluorescence Biodistribution

Immediately following imaging, a cohort of mice were sacrificed (*n* = 3 per group), organs excised, and imaged in reflectance mode using the FMT 2500. The mean fluorescence intensity was measured after drawing an ROI around each organ. Data are expressed as mean fluorescence (in counts/energy) reported as means ± S.E.M.

### 2.13. Tissue Localization

Immediately following *in vivo* imaging, mice were sacrificed, the intact lungs excised, perfused with OCT, and snap frozen in OCT. Ten micron sections were prepared and imaged using fluorescence microscopy with appropriate filters (Zeiss Axioskop 2 MOT Plus). DAPI was used as a nuclear counterstain. Comparable sections were stained with Hematoxylin and Eosin using standard protocols (Mass Histology, Worcester, MA).

### 2.14. Statistical Analysis

Data are presented as the means ± S.E.M. Significance analysis of differences between groups was conducted using a two-tailed unpaired Student's *t-*test when comparing healthy controls and LPS/fMLP groups or ANOVA when 3 or more groups were compared (StatView, SAS Institute). Probability values of <5% were considered significant.

## 3. Results

### 3.1. Characterization of the Activatable NIR Fluorescent Imaging Agent NE680

**Figure 1:**
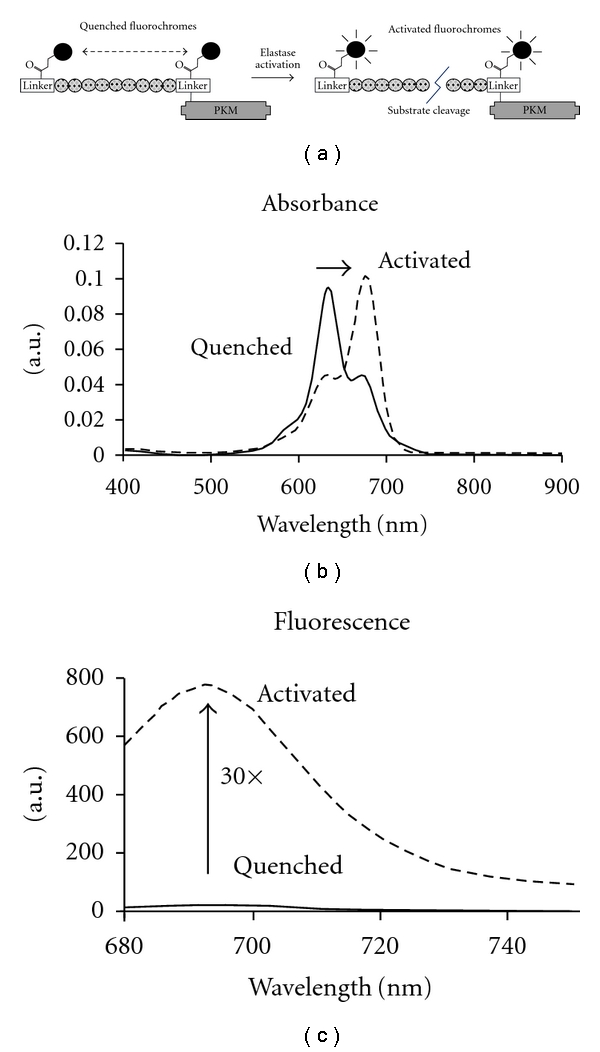
Chemical design and properties of NE680. (a) The fluorogenic peptide substrate is conjugated to a pharmacokinetic modifier (PKM) and flanked by two NIR fluorophores. Upon cleavage of the peptide by NE, the fluorophores become fluorescent. (b) Absorbance spectra of the NE-activated fluorescent form (dashed line) shows a bathochromic shift in the absorbance maximum relative to the native autoquenched state (solid line). (c) The fluorescence emission is increased more than 30-fold upon proteolytic activation with a maximum at 690 nm (excitation at 665 nm).

Figure 1(a) provides a schematic of the NE sensing agent, NE680. The construct was built upon a highly specific NE substrate peptide sequence PMAVVQSVP flanked by two VivoTag-S680 NIR fluorochromes. This sequence has been shown to be highly selective for mouse and human NE over mouse PR3. The discrete pair of closely associated fluorochromes is about 96–98% self-quenched, reflecting static, groundstate quenching in the native state, and exhibits a hypsochromic shift in absorbance of about 50 nm (Figure 1(b)). The substrate was further conjugated to a pharmacokinetic modifier (PKM) comprising an amphiphilic polymer to optimize the rate of blood clearance (Figure 1(a)). Upon proteolysis of NE680 by NE, the quenched fluorochromes become highly fluorescent, 30-fold higher than background (Figure 1(c)). The doubly labeled peptide was characterized by LCMS, UV-Vis and fluorescence spectroscopy prior to conjugation to the PKM. Absorbance and emission spectra of the autoquenched and enzyme-activated forms are shown in Figure 1(c). Incubation for 24 h in mouse plasma at 37°C confirmed that the agent is optically stable and remains quenched in a biological environment (data not shown). The half-life of NE680 in plasma, measured by collecting blood samples at various time points after *i.v.* injection of the agent and analysis by HPLC, was calculated to be *t*_1/2_ = 3 h (data not shown).

### 3.2. NE680 is Preferentially Cleaved by Neutrophil Elastase

**Figure 2:**
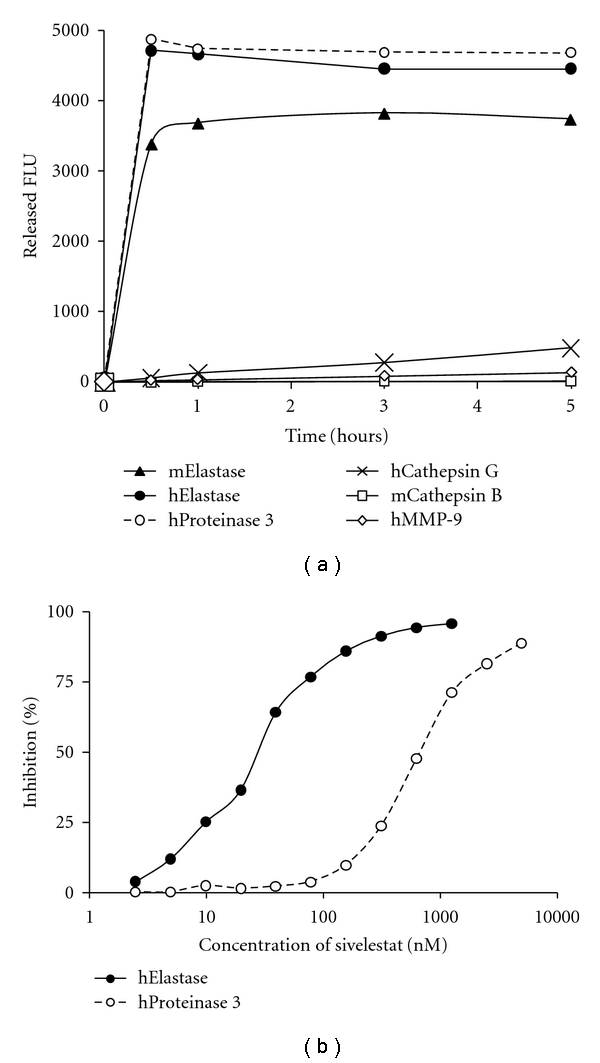
Activation of NE680 by NE and effect of sivelestat. (a) The agent (0.5 *μ*M) was activated *in vitro* by a panel of enzymes (0.05 *μ*M) in optimized buffers and pH for each enzyme and the fluorescence monitored up to 5 h in a fluorescence microplate reader. Released fluorescence was obtained by subtracting the fluorescence of NE680 agent only from that of the NE680 in the presence of enzymes. (b) Inhibition of human NE or PR3 by sivelestat. Reactions were carried out in the presence of varying concentrations of sivelestat. The IC_50_ represents the concentration of sivelestat needed to achieve 50% inhibition of NE680 activation.

To establish the enzyme selectivity of the agent, we compared the activation of NE680 by both human and mouse NE and a variety of closely related enzymes Cat G and PR3, as well as MMP-9 and Cat B which are abundant proteases in neutrophils. *In vitro*, NE680 was more rapidly and selectively activated by human and mouse NE and human PR3 than by mouse Cat B, human Cat G and MMP-9 (Figure 2(a)). At 5 h, cleavage by mouse NE was over 250-, 30-, and 10-fold higher than by Cat B, MMP-9, and Cat G, respectively. To distinguish NE activity from that of PR3 in subsequent studies, the IC_50_ of sivelestat on NE680 activation was determined for the two enzymes. NE was inhibited in a dose-dependent fashion by sivelestat with an IC_50_ of 30 nM while its inhibition of human PR3 was significantly less potent with an IC_50_ of 700 nM (Figure 2(b)).

### 3.3. Experimental Acute Lung Injury

**Figure 3:**
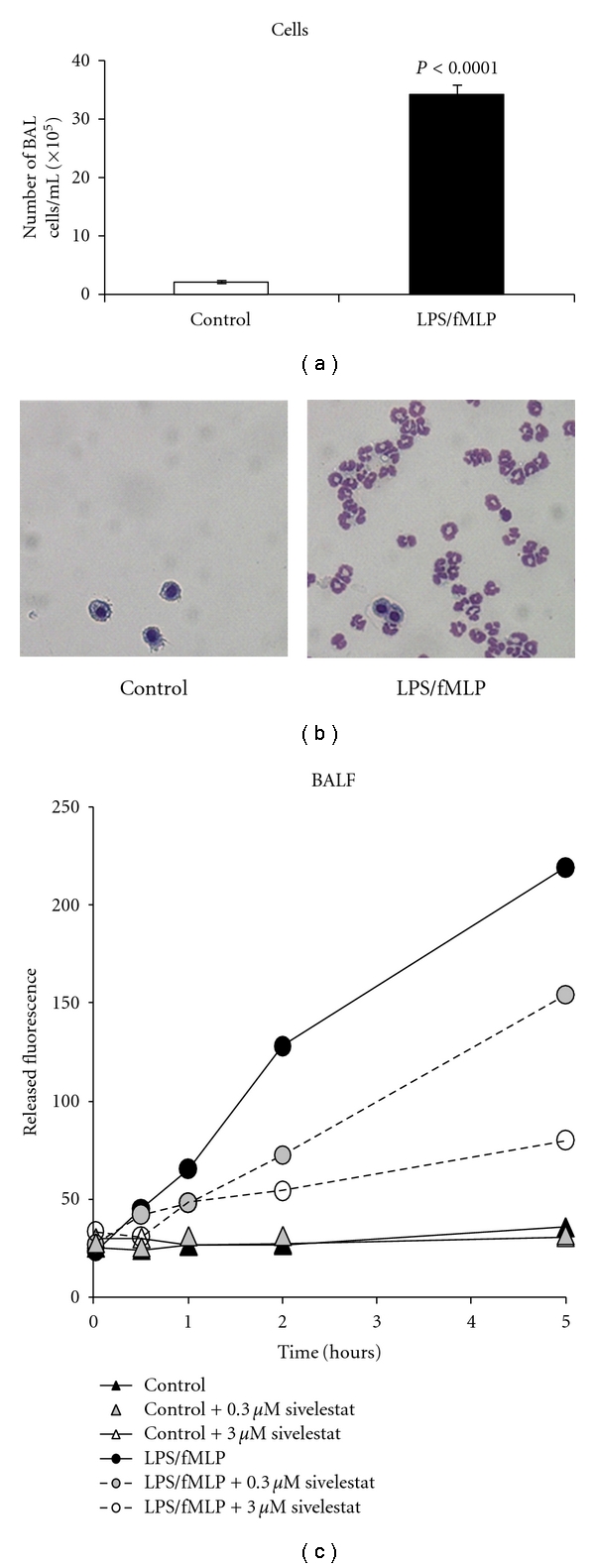
Bronchoalveolar cellular infiltration 24 h after LPS challenge. Mice were challenged i.n. with 100 *μ*g of LPS followed 18 h later by fMLP (200 nM in 40 *μ*L PBS). Five hours later, mice were sacrificed, and bronchoalveolar lavage collected. (a) Cells were counted using a hemocytometer. Data is shown as means ± S.E.M. (*n* = 5 mice per group) of a representative experiment. (b) Cells were spun unto glass slides, stained with Giemsa and observed under microscopy. Shown are representative images from a control mouse and a mouse with ALI. Note the presence of numerous neutrophils in the BAL of the ALI mouse. (c) NE activity in the bronchoalveolar lavage fluid (BALF) was measured using the MeOSu-AAPV-AMC fluorometric substrate. Reactions were monitored at various time points at excitation/emission wavelengths of 400/505 nm using a fluorescence microplate reader. Shown is the released fluorescence after subtracting background fluorescence of the substrate only.

To investigate whether NE680 could be used to image NE activity *in vivo* we first validated an acute lung inflammation model induced in mice by intranasal challenge with LPS followed by fMLP. In mice challenged with LPS/fMLP, the number of BAL cells was significantly higher than in control healthy mice (Figure 3(a), *P* < 0.0001) and over 95% of the cells in LPS/fMLP-induced mice were neutrophils when examined under microscopy (Figure 3(b), right panel). NE activity was quantified in BALF and lung lysates using the commercially available substrate MeOSu-AAPV-AMC. Significantly higher NE activity was detected in BALF (6-fold, *P* = 0.0165, Figure 3(c)) from mice with ALI than controls. Lung lysates from mice with ALI also exhibited higher NE activity (4-fold as compared to lungs from control mice, *P* = 0.0032, data not shown). 

### 3.4. Specific Activation of NE680 in Lung Sections

**Figure 4:**
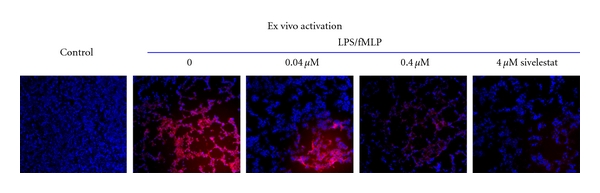
Effect of sivelestat on the activation of NE680 *ex vivo* in lung sections. CD-1 mice were challenged with LPS and fMLP, lungs collected 5 h after fMLP and snap frozen. Vehicle-treated mice served as controls. Lung NE activity was assessed *in situ *by incubating lung sections (10 *μ*m thick) with 1 *μ*M NE680 at 37°C for 5 h in the absence or presence of the NE inhibitor sivelestat. Fluorescent microscopy images were captured with an acquisition time of 2.5 s using a microscope equipped with xenon light source and Cy5.5 filters. Shown are representative images at a final 400× magnification. In blue, DAPI nuclear stain, in red, activated NE agent.

Having measured increases in elastase activity in the lungs and lung extracts and BALF of LPS/fMLP-challenged mice, we determined whether NE680 could be activated *in situ* in lung sections. Thus, 10 *μ*m thick frozen lung sections from control and mice with ALI (not lavaged) were incubated with NE680 for 5 h at 37°C to allow sufficient time for cleavage/activation by secreted tissue proteases. Lungs from mice with ALI showed strong fluorescence, indicating activation of the agent whereas there was little or no apparent fluorescence in control lungs (Figure 4). It should be noted that, when using intranasal instillation, it is technically quite difficult to ensure even distribution of LPS/fMLP across all lung surfaces due to the small volume of instillation in each nostril and the variability of anesthesia effects between mice. Thus, intranasal instillation causes an uneven distribution of neutrophil-mediated inflammation throughout the lung despite careful and slow instillation via both nostrils. Notwithstanding these caveats, the observed activation was suppressed in a dose-dependent manner by increasing concentrations of the NE-selective inhibitor sivelestat, with the higher doses of 0.4 and 4 *μ*M (at doses well below the IC_50_ for human PR3) almost completely blocking NE680 activation. Taken together, the inhibition of NE680 activation at different doses of sivelestat correlated well with the inhibition curves observed in vitro, despite significant experimental differences between the tissue and biochemical assays.

### 3.5. *In Vivo* Real-Time Noninvasive Imaging and Quantification of NE680 Signal Correlates with NE Activity

**Figure 5:**
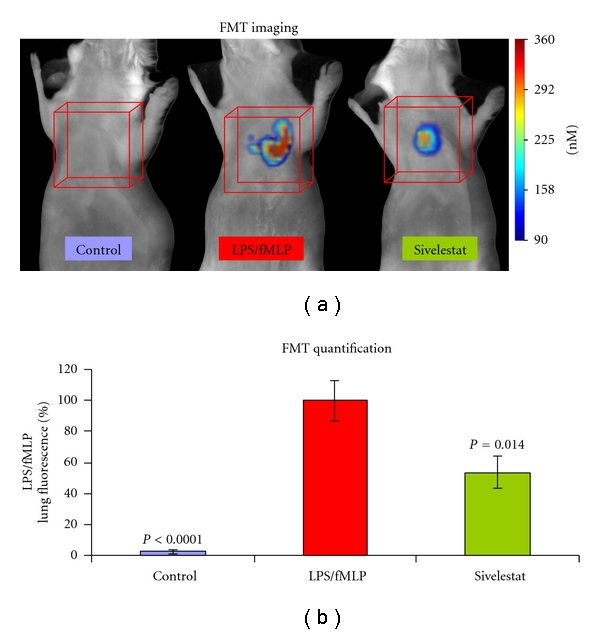
Imaging and quantification of NE680 activation *in vivo*. CD-1 mice were challenged i.n. with LPS and fMLP. A subset of mice was also treated with the NE inhibitor sivelestat 15 min prior to fMLP and NE680 (4 nmoles i.n.) and mice imaged 5 h later by FMT 2500. (a) Representative volume rendering projections taken at the same color gating from control, LPS/fMLP and LPS/fMLP mice which had been treated with sivelestat (5 mg/kg i.n.). (b) The mean concentration of fluorescence (in nM) was quantified in specific ROIs for the lung area in control mice (*N* = 12), mice with ALI (*N* = 16), and Mice with ALI treated with sivelestat (*N* = 12) at a dose of 5 mg/kg i.n.

**Figure 6:**
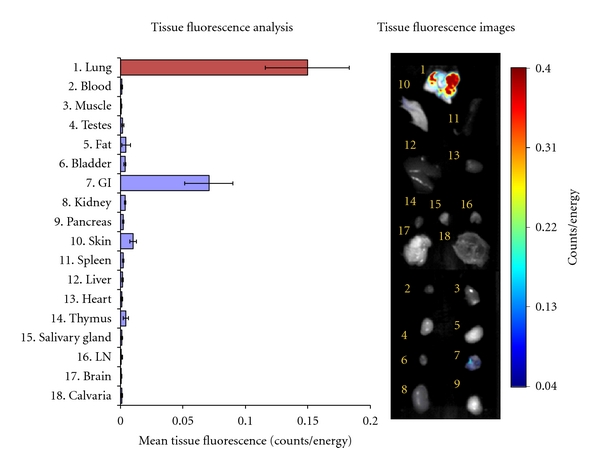
Fluorescence biodistribution of activated NE680. Immediately after imaging, organs from 3 mice challenged with LPS/fMLP were excised and imaged on the FMT 2500 system using the reflectance mode. Regions of interest were drawn around each organ using the FMT software and the mean fluorescence (Counts/Energy) determined. Shown are means ± S.E.M. Insert shows an image of the fluorescence detected in different organs of a representative ALI mouse.

**Figure 7:**
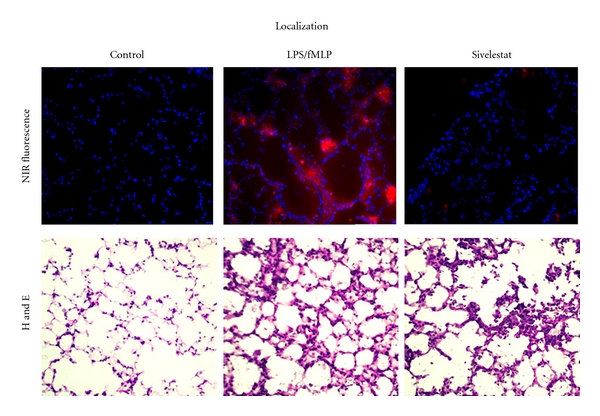
Localization of activated NE680 *ex vivo*. Lungs were snap frozen in OCT for fluorescence microscopy. The distribution of NIR fluorescence was determined using fluorescence microscopy. Digital images were captured using appropriate filters for DAPI, and the near-infrared agent. In the top panel, the distribution of activated NE680 is shown in red, nuclei are counterstained with DAPI (blue). Final magnification 200×. Bottom panel shows comparable sections taken from the same specimens showing increased cell infiltration in the lung of mice with ALI and ALI treated with sivelestat as compared to controls.

The ability of NE680 to be cleaved *in vivo* in a murine model of ALI was substantiated by visualizing and quantifying the NIR signal using quantitative molecular tomography with the FMT 2500. Fluorescence was readily detected in the lung region of all mice with lung inflammation but not in control healthy mice (Figure 5(a)). The fluorescent signal was quantified by drawing 3D ROIs in the chest area, encompassing the lungs and applying a universal threshold equaling 40% of the averaged mean fluorescence of the LPS/fMLP mice. To further assess *in vivo* the role of NE in the activation of NE680, sivelestat was delivered i.n. using a therapeutic dosing regimen initiated 18 h after LPS (after the influx of neutrophils), prior to administration of NE680. The total fluorescence was significantly higher in mice with ALI (*n* = 16; median concentration of 164 nM) as compared to healthy controls (*n* = 12; 0 nM) or mice treated with sivelestat (*n* = 12; 73 nM). To allow comparison between studies varying in the magnitude of signal in positive control animals (range from 30–190 pmols fluorescence/lung), the data from each study was normalized to the average fluorescence in the LPS/fMLP group. The data shown in Figure 5(b) reveals that sivelestat, delivered i.n. just 15 min prior to fMLP and NE680 challenge significantly decreased the activation of the agent in mice with ALI (53.57 ± 10.34% versus 100 ± 13.2%, resp., *P* = 0.0144) while control mice had almost no detectable fluorescence (2.94 ± 1.51% of ALI mice, *P* < 0.0001). These percentages correspond to averages of ~100 pmol fluorescence/lung in LPS/fMLP mice, with control mice showing <5 pmol fluorescence/lung. Fluorescence biodistribution in the excised organs taken immediately after imaging showed significantly higher NE680 signal in the lungs as compared to all other tissues (between 30- to 170-fold higher signal, Figure 6). Cryostat sections of the lungs showed much higher NIR fluorescent signal in the lungs of mice with ALI as compared to either controls or ALI mice treated with sivelestat (Figure 7). Fluorescence appeared to be mostly localized in and around the alveolar walls, the interstitium as well as in the leukocyte infiltrates. A small amount of signal was also found in the alveolar lumen. Neutrophils influx was mostly apparent in the ALI and sivelestat groups while control mice exhibited normal cellularity (Figure 7, bottom panel).

## 4. Discussion

Acute neutrophilic inflammation is a hallmark of acute lung injury and ARDS. Of the various enzymes secreted by neutrophils at the sites of inflammation, NE stands out for its pleiotropic effects. While it plays a beneficial role in innate host defense, unbalanced NE can lead to organ damage and dysfunction [3, 6] and as such has been implicated in a wide range of pathological conditions such as sepsis, emphysema, COPD, and cystic fibrosis, in addition to ALI/ARDS [20]. Given its well-documented deleterious and cytotoxic nature, a specific imaging agent would prove invaluable for understanding the biological functions of NE and the development of NE inhibitors. In this report we describe the use of a newly developed specific NE molecular imaging agent, NE680, to detect and quantify NE activity *in vivo*, in real time and noninvasively in a mouse model of ALI. Because specificity was of key importance, the substrate sequence used to construct the imaging agent was chosen to be rapidly and selectively cleaved by NE while remaining resistant to other proteases including mouse PR3 [16]. Both PR3 and NE are abundant serine proteases (approximately 100 ng PR3/10^6^ cells, 150 ng–3 *μ*g NE/10^6^ cells) [21, 22] with potentially overlapping substrates. In addition, it has been reported that mouse and human PR3 exhibit different species specificities based on the analysis of 3D models [16]. The main difference between mouse and human PR3 was found to reside in the S2 subsite and the fact that mouse PR3 has a more negative net charge than human PR3. Based on these observations which set the precedence for species-specific sequences, NE680 was designed with a high likelihood of preferential cleavage by NE (both mouse and human) and not by mouse PR3. 

The ultimate goal of this study was to be able to visualize and quantify NE activity *in vivo*, in real time and noninvasively. To this end, we first validated a well-known ALI model induced by intranasal challenge with LPS and fMLP which act synergistically to cause lung inflammation characterized by massive neutrophil infiltration and degranulation [18, 19, 23]. As expected, LPS/fMLP administration resulted in a significant increase in the number of BAL cells, particularly neutrophils (Figure 3(a)). The agent designed for NE imaging, NE680, was developed with a specific NE-activatable sequence flanked by fluorochromes placed in close enough proximity to each other for efficient self-quenching of fluorescence. This generated a fluorescent-labeled agent that remains optically silent in the non-activated state but becomes fluorescent upon cleavage of the connecting substrate sequence. Specifically, NIR fluorochromes were used because the NIR spectrum provides maximal tissue penetration and minimal absorption by physiological absorbers such as hemoglobin or water. Modifications of the agent with a PKM extended the plasma and tissue half-lives, allowing NE680 to accumulate and activate in target tissues. Direct delivery of NE680 i.n. to the airspaces of ALI and control mice was performed to facilitate an optimal intraluminal readout of the airspaces. Additional studies using intravenous administration of NE680 also showed effective imaging of inflamed lungs; however, somewhat increased background fluorescence was detected in control lungs, attributed to extrapulmonary degradation/activation and distribution to lung tissue (data not shown). This finding of increased background signal with intravenous injection was unique to pulmonary imaging and did not occur in preliminary studies in wound healing and acute paw edema models (data not shown). 

The fluorescence signal emitting from the lungs was easily detected and quantified using FMT imaging. Three-dimensional ROIs drawn around the lungs allowed calculation of the local concentration in nM and total amount, in pmol, of activated NE680 present. Significantly higher NE680 activation, proportional to increased levels of NE activity (as determined by an independent method, Figure 3(c)), was quantified in the lungs of ALI mice as compared to controls which had barely detectable fluorescence (Figure 5). Excision of tissues following in vivo imaging showed significant fluorescent signal in lungs as assessed by reflectance NIRF imaging. An uneven distribution of neutrophil-mediated inflammation throughout the lung following intranasal instillation was seen despite careful and slow instillation via both nostrils; the gastrointestinal tract also contained fluorescence which is linked to the agent administration technique [24]. Nevertheless, analysis of the lung sections revealed significantly higher fluorescent signal from activated NE680 in the lungs from mice with ALI (Figure 7). It must be noted that lungs were inflated with OCT to avoid collapse during freezing and this could have resulted in BAL cells and fluid (and thus activated NE680) being diluted and/or flushed from the alveolar spaces. Notwithstanding this caveat, H and E sections further confirmed the presence of neutrophils in the inflammatory lung tissue with ALI and revealed that the distribution of activated NE680 fluorescent signal is associated with infiltrated neutrophils within the lung (Figure 7) but is also found in the interstitium and lumen. Neutrophil infiltration was also apparent in the sivelestat group but was absent in the control group as shown on the bottom panels of Figure 7 with H and E staining. Neutrophil influx was not inhibited with this single sivelestat treatment protocol and thus, the readout is truly a mechanistic biomarker for elastase activity (not a composite of effects on enzyme and cell numbers).

To determine whether the activation signal of NE680 that we had quantified *ex vivo* and *in vivo* was due to NE activity alone, a number of corroborating studies had to be performed. *In vitro*, we could not verify the inability of mouse PR3 to cleave NE680 because it is not commercially available. However, the sequence used to construct NE680 is known to be minimally cleaved by mouse PR3 [16]. As an additional verification of selectivity, we determined that the known NE-specific inhibitor sivelestat potently blocked the activation of NE680 by NE (IC_50_ = 30 nM similar to what has been reported previously) [17] with less potency on human PR3 (IC_50_ = 700 nM). This is also in agreement with previous studies showing the higher selectivity of sivelestat towards NE over trypsin, thrombin, plasmin, plasma kallikrein, pancreas kallikrein, chymotrypsin, and Cat G [17]. We used intact lung sections from ALI mice, tissue known to have high numbers of neutrophils and secreted Cat G, NE, and PR3 proteases, to evaluate the activation and specificity of NE680. In such *ex vivo* studies, NE680 fluorescence was significantly inhibited by as little as 40 nM sivelestat (Figure 4). Since the agent's cleavage site is the NE-specific substrate PMAVVQSVP [16] and the IC_50_ of NE over human PR3 is 23-times lower, it is very likely that the dominant neutrophil protease involved in NE680 activation is NE, and likely not PR3 or other off-target proteases associated with lung inflammation. Other studies, using lung tissue homogenates were inconclusive and required high doses of sivelestat to inhibit either NE680 or AAPV-AMC cleavage (data not shown), suggesting that the homogenization procedure released unrelated tissue intracellular proteases not normally seen extracellularly in ALI.

Inhibition of NE activity *in vivo* was also achieved by treatment with sivelestat, in agreement with previous reports on its efficacy in animal models of lung injury [25–29]. It is important to note that in these and other reports, sivelestat was administered before or at the time of the challenge (LPS, ventilator-induced injury, and pneumococcal pneumonia), and under these conditions sivelestat also inhibited the migration of neutrophils into the site of injury. As a result, it is difficult to dissect the effect of sivelestat on NE from the effect on neutrophil recruitment. In our studies, to better demonstrate that sivelestat can directly inhibit NE activity, and thus NE680 activation, the inhibitor was delivered 18 h after LPS challenge at a time when neutrophil migration had already occurred. Thus, it can be inferred that the significant, although partial, inhibition of NE680 activation observed *in vivo* (Figure 5(b)) is the direct result of the inhibition of NE activity. The inability to completely block NE680 activation *in vivo* is most likely due to the inefficiency of sivelestat delivery and target coverage *via* i.n. administration, as nearly complete inhibition was achieved in ALI lung frozen tissue sections with as little as 40 nM sivelestat. Taken together, these interventional studies provide strong evidence that NE activity in acute lung inflammation can be quantified and monitored *in vivo*, in real time, and noninvasively.

## 5. Conclusion

In summary, we have developed a selective NE sensitive fluorescence activatable agent, NE680, and demonstrated its ability to image and quantify NE activity noninvasively in a model of ALI. To our knowledge, this is the first molecular NE imaging agent that can quantify increased NE activity in lung inflammation and the efficacy of selective therapy *in vivo*. The combined in vitro and in vivo properties of NE680 and efficacy in imaging LPS/fMLP-induced ALI suggest it may be useful in other chronic neutrophil-driven disease models, such as emphysema/chronic obstructive pulmonary disease, cystic fibrosis, acute neutrophilia, chronic wound healing, rheumatoid arthritis, and infectious diseases. In such models, which do not use fMLP to induce neutrophil degranulation, the detection of the spontaneous release of NE during inflammation may reveal subtleties in onset and overall kinetics that may further enhance our understanding of these diseases. Furthermore, by quantifying elastase-mediated molecular processes, we believe that such a highly specific agent combined with tomographic imaging will help in the development of novel pharmacological interventions *in vivo*. Application of such an agent in the context of NIRF bronchoscopy would prove a valuable addition to conventional imaging tools [30] in diagnosing, staging, and monitoring of patients with lung inflammatory diseases or cancer.
